# Spontaneous Bilateral Vesical Artery Pseudoaneurysms Presenting With Massive Recurrent Hematuria and Obstructive Acute Kidney Injury: A Case Report

**DOI:** 10.1002/ccr3.73739

**Published:** 2026-10-16

**Authors:** Dheeraj Kumar Alias Sailish, Amisha Kumari, Hadia Jamil, Sardar Ravi Singh, Muhammad Hassaan Javaid, Muddassir Khalid, Fred Segawa

**Affiliations:** ^1^ Ghulam Muhammad Mahar Medical College Sukkur Pakistan; ^2^ Niazi Welfare Teaching Hospital Sargodha Pakistan; ^3^ Liaquat University of Medical and Health Sciences Jamshoro Pakistan; ^4^ Shifa College of Medicine Islamabad Pakistan; ^5^ Nishtar Medical University, Nishtar Hospital Multan Multan Pakistan; ^6^ Makerere University College of Health Sciences Kampala Central Region Uganda

**Keywords:** acute kidney injury, angioembolization, clot retention, hematuria, hepatitis B, hepatitis D, interferon‐alpha, pseudoaneurysm, vesical artery

## Abstract

Spontaneous development of vesical artery pseudoaneurysm (VA‐PA) as a cause of life‐threatening hematuria is very rare. Most of the existing reports are due to trauma or other types of pelvic surgeries/urological procedures. Incomplete hemostasis in these patients can lead to subsequent complications such as clot retention, severe blood loss resulting in anemia and obstructive AKI. We report this extremely rare case of a 35 year old lady with bilateral VA‐PA causing recurrent episodes of massive hematuria and obstructive AKI that was ultimately resolved by selective embolization. A 35‐year‐old female presented with a history of gross hematuria along with clot retention and severe anemia. She also had signs of obstructive AKI which required her to have temporary dialysis. Cystoscopy showed intravesical clot but no bleed site was identified on initial evaluation. On contrast‐enhanced CT scan, there were pseudoaneurysms found from both sides coming off from left superior and right inferior branches of vesical artery. Following confirmation by selective pelvic angiogram, we did a superselective embolization using micro coils and PVOH particles to occlude both pseudoaneurysms. This resulted in immediate cessation of hematuria, normalization of renal functions and stabilization of Hb values. At last follow‐up done in 6 weeks' time, all symptoms had resolved and there was complete thrombosis of both the lesions seen on imaging. Bilateral spontaneous vesical artery pseudoaneurysm is a condition that must be suspected in a patient presenting with recurrent episodes of severe hematuria despite having undergone seemingly successful endoscopic treatment. Immediate imaging of the vessels and selective angioembolization is the surest means of diagnosis and treatment, thus avoiding further complications.

## Key Clinical Message

Persistent severe hematuria even after apparent resolution of clots needs to prompt the clinician for a vascular etiology. Early intervention with CT angiography and angiembolization can be life‐saving in cases of spontaneous pseudoaneurysm of the vesical artery.

## 1 Introduction

Gross hematuria is a common presentation in urology, with etiologies varying from UTIs and urolithiasis to malignancy, coagulopathy, or vascular disorder. Gross hematuria in such volume as to cause acute kidney injury and severe anemia is rare, usually indicative of postrenal obstruction or massive bleeding from vessels which is not possible to compensate by physiologic mechanisms. A pseudoaneurysm, or false aneurysm, develops due to an arterial rupture, with an extraluminal hematoma connected with the source vessel. It differs from a true aneurysm in the structure of its walls; in a pseudoaneurysm, it consists of organized thrombus and connective tissue [1].

Pseudoaneurysms involving the vesical or pelvic vasculature are frequently iatrogenic and/or posttraumatic in nature, secondary to pelvic surgery, radiation therapy, transurethral interventions, or catheter placement [2, 3, 4]. Spontaneous pseudoaneurysm, which occurs independently of any preceding precipitating instrumentation or trauma, is a very uncommon entity, with only sporadic case reports in the literature [2, 3].

The current case is presented based on the CARE (CAse REport) criteria for transparent case report publication [5]. We present the case of a spontaneous vesical pseudoaneurysm in a young woman, with the presentation of recurrent hemorrhagic cystitis with anemia, clot retention, and acute renal dysfunction, which was managed with selective angiographic embolization, and compare it to other similar cases previously described in the literature.

## 2 Case History

A 35 year old woman, with no significant past medical, surgical, or obstetric history, developed a 24 h history of gross hematuria, generalized weakness, and decreased urine outflow. There were no reports of trauma, pelvic instrumentation, urinary catheterization, anticoagulant usage, or prior episodes of hematuria on her part.

Her menstrual history was also unremarkable with regular cyclicity. On admission, this patient appeared pale and was found to have a heart rate of 112 bpm. Despite this, there was no evidence of any hemodynamic instability as indicated by the patient's blood pressure reading of 110/70 mmHg. Mild suprapubic tenderness was detected along with the presence of a palpable, distended urinary bladder on physical examination, but there was no sign of peritonitis.

### 2.1 Differential Diagnosis

A number of other possible reasons for massive repeated hematuria were examined prior to making the diagnosis.

- Bladder neoplasia, primarily urothelial carcinoma, was ruled out by urine cytology and repeated cystoscopic biopsies, showing acute and chronic inflammation with localized hemorrhages but no dysplastic or malignant changes.
- Vascular anomalies, namely congenital arteriovenous malformation and arterio‐vesical fistula, were excluded by multiphasic CT angiography, which revealed saccular pseudoaneurysms without any vascular nidus and early venous filling.
- Systemic coagulopathy associated with chronic hepatitis B/hepatitis D coinfection and interferon treatment was one of the possibilities. Coagulation analysis did show thrombocytopenia and coagulation disorders, but they could contribute only to the extent of bleeding and were unlikely to be responsible for the localized arterial bleeding. The diagnosis was finally made by selective pelvic angiography, which showed vesical artery pseudoaneurysms bilaterally and allowed successful endovascular embolization.

### 2.2 Investigations

On initial evaluation at the hospital, the patient was found to have a severe normocytic normochromic anemia (hemoglobin [Hb] level = 5.3 g/dL), thrombocytopenia (platelet count = 92 × 10^3^/μL), and severe AKI caused by urinary outflow obstruction. Serum creatinine levels were 6.8 mg/dL, BUN was 84 mg/dL, and eGFR was 7.4 mL/min/1.73 m^2^. Mild coagulopathy was noted in terms of prolonged PT (prothrombin time) and INR values as follows: PT‐14.2 s; INR‐1.25 (Table 1).

**TABLE 1:** Summary of baseline investigations.

Parameter	Patient value	Reference range
Hemoglobin	5.3 g/dL	12.0–15.5 g/dL
Serum creatinine	6.8 mg/dL	0.6–1.1 mg/dL
Blood urea nitrogen (BUN)	84 mg/dL	7–20 mg/dL
eGFR	7.4 mL/min/1.73 m^2^	> 90 mL/min/1.73 m^2^
Platelet count	92 × 10^3^/μL	150–450 × 10^3^/μL
Prothrombin time (PT)	14.2 s	11.0–13.5 s
INR	1.25	0.8–1.1

Her urine analysis showed haematuria. After admission at our hospital she had renal and pelvic ultrasonography revealing bilateral normal‐sized kidneys without hydronephrosis but marked distention of her urinary bladder with presence of a large heterogeneous non‐shadowing intravesical mass measuring 9.4 × 7.2 cm which correlated with findings of extensive clot retention.

Her renal function was deteriorating despite treatment for suspected urinary tract infection; hence, she underwent urgent hemodialysis twice due to progressive renal failure and metabolic acidosis via temporary right internal jugular venous catheter as well. Emergency rigid cystoscopy after metabolic stabilization revealed a huge extent of intra‐vesical blood clots that were removed via ellick evacuator and diffuse mucosal ooze hindered view to find a discrete source of bleeding or structural lesion.

### 2.3 Management

Insertion of 22 Fr three‐way foley catheter followed by continuous irrigation of bladder with normal saline resulted in resolution of hematuria with clearness of irrigation fluid. Patient's serum creatinine level decreased from initial 4.0 to 1.4 mg/dL and was discharged from hospital after 48 h. Within 48 h of discharge, she again developed recurrent severe gross hematuria and passage of big size blood clot along with profound lethargy. Recurrent severe anemia was detected and the patient was transfused three unit pack red cell. Due to concern regarding secondary UTI/post‐procedural bacteremia, Intravenous Ceftriaxone (1 g every 12 h) was started empirically.

In order to determine the site of hemorrhage, a multiphasic CT urogram with contrast was conducted in an urgent manner. Arterial phase images showed two areas of hyperenhancement which corresponded to vesical artery pseudoaneurysms; one was a 1.8 × 1.4 cm pseudoaneurysm of the left superior vesical artery, while the other was a 1.1 × 0.9 cm pseudoaneurysm of the right inferior vesical artery. The presence of synchronous arterial enhancement with persistent delayed contrast filling was evidence of bilateral urinary bladder pseudoaneurysms.

Upon further review of the patient's history, it was found that the patient had been diagnosed with chronic hepatitis B virus and hepatitis D virus infection three months ago, receiving weekly subcutaneous injections of pegylated interferon α‐2a (180 μg) under gastroenterology follow‐up. Initial coagulation parameters were within normal limits prior to therapy. However, laboratory tests conducted a week before admission had shown thrombocytopenia with moderate PT/INR prolongation due to myelosuppression secondary to interferon therapy.

After multidisciplinary discussion, the patient was subjected to emergent endovascular embolization. Right common femoral arterial access was achieved under local anesthesia, and both sides of internal iliac arteries were selectively catheterized via a 5‐Fr Roberts uterine catheter. Left superior vesical artery and right inferior vesical artery were then superselectively catheterized via a 2.8‐Fr Progreat microcatheter. Pseudoaneurysm on the left side was embolized by two 3 mm × 4 cm pushable microcoils, while the right side feeding branches were embolized using 300–500 μm polyvinyl alcohol particles. Completion angiography revealed successful exclusion of both pseudoaneurysms with adequate perfusion of the other pelvic vessels.

Different embolic agents were selected for the two lesions based on differences in feeding‐vessel anatomy and the microcatheter position that could be safely achieved. The left superior vesical artery pseudoaneurysm was supplied by a single, short, relatively straight feeding branch that allowed the microcatheter tip to be advanced close to the aneurysm neck; this anatomy favored precise, mechanical sac occlusion with pushable microcoils and permitted immediate angiographic confirmation of stasis. The right inferior vesical artery pseudoaneurysm, by contrast, arose from a more distal, smaller‐caliber branch pattern that limited how close the microcatheter could be positioned to the aneurysm neck; a particulate agent was therefore chosen to occlude the small terminal feeding vessels supplying the pseudoaneurysm while minimizing the risk of coil migration or reflux from a less stable catheter position. This combined, anatomy‐tailored approach allowed durable exclusion of both lesions while preserving perfusion to adjacent pelvic structures.

Selective pelvic angiography performed through right common femoral arterial access confirmed the CTA findings and demonstrated distinct vascular anatomy on each side. The left superior vesical artery pseudoaneurysm measured 1.8 cm and arose from a short, relatively straight, larger‐caliber feeding vessel. This anatomy allowed the 2.8‐Fr Progreat microcatheter to be advanced safely close to the pseudoaneurysm neck, permitting precise deployment of two 3 × 4 cm pushable microcoils across the neck and within the feeding vessel, resulting in immediate mechanical occlusion. In contrast, the 1.1‐cm right inferior vesical artery pseudoaneurysm was supplied by a distal, narrow‐caliber, tortuous branch that limited safe microcatheter advancement to the lesion neck. Therefore, 300–500 μm polyvinyl alcohol (PVA) particles were selected to achieve distal occlusion of the terminal feeding arterial branches while minimizing the risk of coil migration or reflux from a less stable catheter position. Completion angiography demonstrated complete exclusion of both pseudoaneurysms with preservation of flow within the major pelvic arterial circulation.

### 2.4 Outcomes and Follow‐Up

The gross hematuria completely subsided within 12 h after embolization procedure. Urinary catheter was withdrawn on the second post‐operative day, and the patient voided urine clearly without any difficulty. Hemoglobin concentration was stable at 10.1 g/dL pre‐discharge, and the renal function recovered, with serum creatinine decreased to 0.8 mg/dL.

At six‐week follow‐up visit, the patient was asymptomatic without hematuria and lower urinary tract symptoms recurrence. Pelvic ultrasonography and CT angiography showed complete thrombosis and regression of both pseudoaneurysms, unremarkable renal morphology and function, and absence of intravesical clot formation.

## 3 Discussion

Hematuria with clot retention leading to acute kidney injury is a rare urological condition but it is also quite serious. The most common cause of such a condition is post‐renal obstruction, where clot leads to increased pressure in the bladder and in the renal pelvis, and therefore decreases transglomerular filtration gradient leading to obstructive AKI [6] and is defined by KDIGO criteria [7]. Such AKI is usually quite reversible when the obstruction is eliminated, which can be seen from improvement in the patient's renal function after clot removal before bleeding occurred again.

Vesical and pelvic pseudoaneurysms are rare vascular abnormalities typically associated with an arterial wall disruption as a result of trauma, pelvic surgeries, radiation, or urological procedures [1]. Predisposing factors for the development of this disease may include transurethral electrocoagulation [2], placement of a suprapubic catheter [3] or, very rarely, rupturing of an adjoining femoral or iliac pseudoaneurysm into the bladder [4]. All published cases show a common clinical course characterized by recurrent pulsatile hematuria, which seems to respond to conservative or endoscopic treatment methods, such as irrigation, electrocautery, and removal of clots, only to start bleeding again, similar to what happened in our current case. There are some articles where the use of electrocautery in the treatment of pulsatile bleeding from the vesica was especially discouraged due to the fact that this method does not correct the underlying vascular lesion but rather aggravates it [2], emphasizing the significance of having a low threshold for performing either cross‐sectional imaging or catheter angiography in case bleeding recurs despite seemingly satisfactory endoscopic control. Likewise, another case series of true spontaneous, non‐traumatic rupture of a pseudoaneurysm, in the absence of any prior manipulation, in the renal artery, even in a young patient on dialysis, has also been reported [8], making the possibility of true spontaneous rupture in certain cases credible.

No antecedent instrumentation, trauma, or pelvic interventions were identified, classifying our lesion within the small subset of spontaneously occurring “vesical” pseudoaneurysms described in the literature.

Coincidental with our case was the diagnosis of chronic hepatitis B virus infection (HBV) that had evolved into a superimposed coinfection with HCV‐D, which was managed with interferon‐alpha. Since the patient was taking pegylated interferon‐a for the treatment of chronic hepatitis B/D infection, no causal link could be made between the use of interferon and the development of pseudoaneurysms due to the sole observation in the patient. Thrombocytopenia and abnormal coagulation profiles were present and likely worsened the bleeding; however, they do not explain the development of bilateral arterial pseudoaneurysms [8]. Significantly, the lack of evidence of symptoms of systemic vasculitis and p‐ANCA, c‐ANCA, ANA, anti‐dsDNA, cryoglobulin, and complement levels made an obvious interferon‐associated systemic vasculitis unlikely. We believe that pseudoaneurysms developed spontaneously and both the interferon use and the infection are only possible contributing factors.

Although most widely documented in the hepatitis C treatment literature, with cases of interferon‐alpha precipitating either new‐onset vasculitis or exacerbating previously existing cryoglobulinemic and polyarteritis nodosa‐like vasculitis being well described [9], interferon alpha‐related leukocytoclastic vasculitis has even been reported in a non‐cryoglobulinemic patient with hepatitis‐B infection [10]. Since coinfection with HCV‐D leads to more severe and accelerated liver disease than HBV alone, and interferon‐based regimens represent the only approved therapeutic strategy for chronic hepatitis delta despite their low rate of achieving a sustained virological response [11], a large proportion of HBV/HDV‐coinfected individuals worldwide (an estimated twelve million people are affected by HDV globally) [12] receive prolonged courses of interferon α. It remains unclear whether the development of a pseudoaneurysm in this individual can be attributed to interference with hepatic blood supply through the actions of interferon α, the underlying viral hepatitis, or another idiopathic entity, yet we offer this observation for its potential to serve as hypothesis generating.

Selective angioembolization is considered the gold‐standard minimally invasive approach to vascular causes of hematuria, such as vesical pseudoaneurysm and arteriovesical fistula [1, 2, 3, 4].

Despite limited experience with transcatheter arterial embolization for intractable bladder hemorrhage (predominantly due to pelvic malignancy instead of pseudoaneurysm), larger case series have demonstrated high (greater than 90%) technical success rates and durable control of hematuria by use of superselective particulate or coil/glue embolization techniques of the vesical arteries [13, 14, 15]. This compares favorably with traditional approaches involving open surgical ligation/resection of affected vessels, which are associated with higher procedural morbidity while preserving bladder/pelvic structures without benefit over embolization in terms of controlling bleeding [3, 6].

## 4 Conclusion

Spontaneous vesical pseudoaneurysm is an unusual entity that may be responsible for recurrent hematuria with potentially fatal sequelae if left untreated. Hematuria recalcitrant to apparently successful clot removal mandates a high index of suspicion when no other sources are apparent. Contrast‐enhanced imaging and selective embolization should be performed as soon as possible once the diagnosis has been made in order to achieve definitive hemostasis without requiring open surgery and subsequent morbidities related to rebleeding and progressive renal damage secondary to urinary tract obstruction.

## Author Contributions

**Dheeraj Kumar Alias Sailish:** conceptualization, methodology, software, investigation. **Amisha Kumari:** writing – original draft, data curation. **Hadia Jamil:** writing – original draft, data curation. **Sardar Ravi Singh:** writing – original draft, data curation. **Muhammad Hassaan Javaid:** writing – review and editing, writing – original draft. **Muddassir Khalid:** writing – review and editing, supervision, project administration. **Fred Segawa:** resources.

## Funding

The authors have nothing to report.

## Ethics Statement

The authors have nothing to report.

## Consent

Written informed consent was obtained from the patient for publication of this case report and accompanying images. A copy of the written consent is available for review by the Editor‐in‐Chief of this journal upon request.

## Conflicts of Interest

The authors declare no conflicts of interest.

## Data Availability

Data are available on request from the corresponding author.
